# From bedside to bench: regulation of host factors in SARS-CoV-2 infection

**DOI:** 10.1038/s12276-021-00595-x

**Published:** 2021-04-07

**Authors:** Samantha Y. Q. Ong, Ilya M. Flyamer, Wendy A. Bickmore, Simon C. Biddie

**Affiliations:** 1 Intensive Care Medicine, NHS Lothian, Edinburgh, UK; 2 MRC Human Genetics Unit, Institute of Genetics and Molecular Medicine, The University of Edinburgh, Edinburgh, EH4 2XU UK

**Keywords:** Chromatin structure, Transcriptional regulatory elements, Transcriptomics, Viral infection, Genetics research

## Abstract

The zoonotic coronavirus SARS-CoV-2 (severe acute respiratory syndrome coronavirus-2), which causes COVID-19 (coronavirus disease-2019), has resulted in a pandemic. This has led to an urgent need to understand the molecular determinants of SARS-CoV-2 infection, factors associated with COVID-19 heterogeneity and severity, and therapeutic options for these patients. In this review, we discuss the role of host factors in SARS-CoV-2 infection and describe variations in host factor expression as mechanisms underlying the symptoms and severity of COVID-19. We focus on two host factors, angiotensin-converting enzyme 2 (ACE2) and transmembrane serine protease 2 (TMPRSS2), implicated in SARS-CoV-2 infection. We also discuss genetic variants associated with COVID-19 severity revealed in selected patients and based on genome-wide association studies (GWASs). Furthermore, we highlight important advances in cell and chromatin biology, such as single-cell RNA and chromatin sequencing and chromosomal conformation assays, as methods that may aid in the discovery of viral–host interactions in COVID-19. Understanding how regulation of host factor genes varies in physiological and pathological states might explain the heterogeneity observed in SARS-CoV-2 infection, help identify pathways for therapeutic development, and identify patients most likely to progress to severe COVID-19.

## SARS-CoV-2: More virus-host interaction studies needed

Aberrant host immune responses probably influence the severity of COVID-19 more than viral activity itself, but more research is needed to understand how the virus and host interact. The effects of SARS-CoV-2 infection vary widely in severity, from asymptomatic cases to multiple organ failure. Simon Biddie at the University of Edinburgh, UK, and co-workers reviewed current knowledge of host factors in SARS-CoV-2 infection. They focused on host enzymes called ACE2 and TMPRSS2, which facilitate viral entry and replication. High expression levels of these enzymes in certain organs may influence disease severity, and genetic variations influencing their expression should be explored. The researchers believe that pathways involving proinflammatory factors also play wide-ranging roles in COVID-19. Advances in sequencing technologies, high-throughput assays and single-cell methodologies will aid exploration of virus-host interactions.

## Introduction

The emergence of the novel coronavirus (CoV) severe acute respiratory syndrome (SARS) coronavirus 2 (SARS-CoV-2) has resulted in a pandemic. In general, the heterogenous symptomatology following SARS-CoV-2 infection, which ranges from asymptomatic to severe life-threatening COVID-19, suggests the involvement of host factors. Epidemiological studies to date have implicated several host factors in severe COVID-19, including male sex, older age, cardiovascular disease, obesity, and diabetes^1,2^. These host factors have behavioral, environmental, and genetic contributions^3^.

Sequencing of the SARS-CoV-2 genome has enabled comparative studies with other beta-coronaviruses to identify pathogenic features. For example, the structural similarity of the SARS-CoV-2 and SARS-CoV spike (S) proteins has accelerated the identification of angiotensin-converting enzyme 2 (ACE2) as a key host factor for viral entry^4^. The endogenous role of ACE2 is to hydrolyse the vasoconstrictor peptide angiotensin. Attachment of SARS-CoV-2 to host cells is mediated by binding of the viral S1 subunit receptor-binding domain to ACE2, permitting entry and replication^5^. Following ACE2 engagement, proteolytic cleavage of S2 by host proteases, such as plasma membrane-associated type II transmembrane serine protease (TMPRSS2), mediates membrane fusion and cytosolic release of the viral RNA. TMPRSS2 is essential for the establishment of airway infection by SARS-CoV-2 and other zoonotic and human CoVs^5^.

In general, understanding host factor regulation through chromatin structure and DNA-binding factor analyses in various cell types and pathophysiological states is crucial. In this review, we discuss the transcriptional regulation of host factors associated with SARS-CoV-2 and severe COVID-19. We focus on key host factors for SARS-CoV-2 infection, namely, ACE2 and TMPRSS2, and examine their expression in cell types linked to symptoms. We also explore regulatory control and context-dependent changes in host factor expression associated with infection and risk phenotypes. Furthermore, we review genetic variants that might confer an increased risk of severe COVID-19 and outline methodologies to guide genotype-phenotype correlation for functional validation of host factors.

## ACE2 and TMPRSS2 tissue distribution and correlation with clinical presentation

**Fig. 1:**
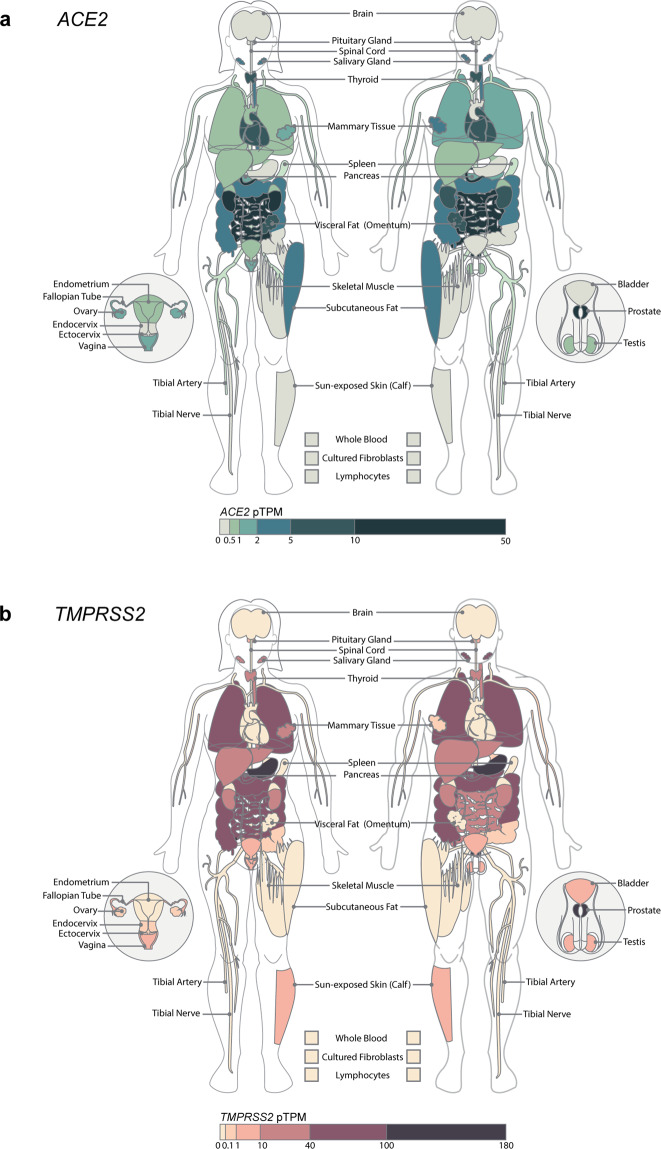
*ACE2* and *TMPRSS2* mRNA human tissue expression. Representation of RNA-seq-derived median protein-transcripts per million (pTPM) levels of *ACE2* (**a**) and *TMPRSS2* (**b**) genes in 54 nondiseased tissue sites in females (left) and males (right). Organs not labeled include the brain, aorta, coronary artery, heart, lungs, liver, esophagus, stomach, terminal ileum, colon (transverse, sigmoid), adrenal glands, kidney, and bladder. Data were obtained from Genotype-Tissue Expression (GTEx) Portal V8^107^.

High-throughput sequencing of RNA (RNA-seq) has shown ubiquitous expression of *ACE2* and *TMPRSS2*. *ACE2* expression is particularly enriched in the lungs, heart, kidneys, intestine, brain, skin, oral mucosa, and endothelial cells^6^ (Fig. 1a). *TMPRSS2* is mainly expressed in the prostate, salivary glands, and luminal epithelia of the gastrointestinal, urogenital and respiratory tracts^7^ (Fig. 1b). Patterns of *ACE2* expression correlate with susceptibility to infection with SARS-CoV^8^, leading to the hypothesis of higher SARS-CoV-2 tropism for tissues with higher *ACE2* expression and consequent symptomatology.

### ACE2 and TMPRSS2 expression and pulmonary pathology

Mainly transmitted through droplets, aerosols, and direct contact, the predominant targets of SARS-CoV-2 are the upper respiratory epithelium and lungs. COVID-19 typically presents with constitutional respiratory symptoms, with the majority of patients having pyrexia and dry cough^9^. The central role of the lung in COVID-19 is supported by a significant rise in SARS-CoV-2 viral load in these organs in more severe disease, which does not occur in stool or serum^10^. In 10-20% of patients, the disease progresses in severity with bilateral ground-glass opacities on chest computed tomography^11^. In the first wave of COVID-19, approximately 5–20% of patients became critically ill, requiring invasive mechanical ventilation due to acute respiratory distress syndrome (ARDS) with bilateral diffuse alveolar damage and cellular exudates on postmortem analysis^12^.

Given their role in SARS-CoV-2 infection, expression of the *ACE2* and *TMPRSS2* genes in the lung may contribute to the observed pathology. Immunohistochemistry demonstrates the highest expression of the ACE2 protein within the sinonasal cavity and pulmonary alveoli, which are presumptive sites of viral transmission and disease development^13^. Single-cell RNA-seq (scRNA-seq) of healthy adult lung tissue from eight donors showed that <1% of cells in the lung parenchyma expressed *ACE2* mRNA, 83% of which were surfactant-producing alveolar type 2 (AT2) cells^14^. A low proportion of *ACE2*^+^ cells has been observed in other scRNA-seq datasets, with proportionally higher A*CE2*^+^ AT2 cells than other cell types^13,15^. Consistent with immunohistochemical evidence of ACE2 expression on apical surfaces^13^, this provides compelling evidence for that the virus targets AT2 cells. scRNA-seq studies have also found that a subset of *ACE2*^+^ cells strongly coexpress *TMPRSS2* and other viral process-related genes, suggesting enhanced vulnerability of AT2 cells to SARS-CoV-2^13,16^. AT2 cells may thus represent a potential viral reservoir for replication or alternatively undergo viral-induced cell death to initiate barrier damage and inflammation.

The proinflammatory state of COVID-19 may also contribute to *ACE2* expression through proinflammatory transcription factor (TF) binding. scRNA-seq of the human lung revealed that *ACE2* expression is associated with expression of interferon-stimulated genes (ISGs) and components of the type I interferon (IFN) signaling pathway, which is central to the immediate antiviral response^17^. In primary human upper airway epithelial cells, stimulation with IFN-γ and IFN-α2 leads to *ACE2* upregulation, possibly through signal transducer and activator of transcription-1 (STAT-1) binding sites in the *ACE2* promoter^17^. Similarly, human goblet secretory cells of the nasal epithelia exhibit ACE2 upregulation in response to influenza viruses, likely via an IFN pathway^17^. IFN-induced ACE2 expression may confer susceptibility to severe disease: local induction of IFN and ISG have been observed in the bronchoalveolar lavage (BAL) of critically ill patients^18^, and high circulating IFN levels have been shown to persist in later stages of severe COVID-19^19^. Nevertheless, IFN-induced expression has been shown to represent a non-functional truncated ACE2 isoform, suggesting that conditions of high IFN levels are unlikely to increase viral S-protein-mediated cellular entry to promote infection^20^. Furthermore, the paucity of IFN production and high viral loads observed in the incubation phase^21,22^ suggest a protective role for IFN in early disease, which is currently being evaluated in clinical trials^23^. The relationship between spatiotemporal kinetics and tissue specificity of IFN responses and host factor expression during SARS-COV-2 infection requires further study.

### Extrapulmonary involvement and host factor expression

Several extrapulmonary manifestations of COVID-19 have been observed, from benign symptoms involving the eyes and gastrointestinal and nasal systems to severe organ failure involving the neurological, cardiac, and renal systems. Diarrhea occurs in approximately 5% of cases^24^, while headache and anosmia have been described as common early symptoms^25^. Regarding severe cases, acute cardiac injury was seen in 12% of patients in a Chinese cohort^26^. Severe acute kidney injury (AKI) requiring renal replacement therapy is common among critically ill patients, affecting 20-40% of those in the ICU^1^.

The severity and multisystem involvement observed in a subset of COVID-19 patients suggest a possible role for viral interaction with extrapulmonary host factors. Whole-tissue *ACE2* mRNA expression and recent scRNA-seq studies reported high *ACE2* expression in cardiac pericytes and cardiomyocytes, epithelial cells of the oral mucosa, ileum, stomach, colon and rectum, proximal renal tubules, and endothelial cells^14,27,28^. Similar to the lung, coexpression of *ACE2* and *TMPRSS2* in corneal, intestinal, and nasal epithelial cells is associated with activation of innate immune pathways, highlighting these cells as having potential roles in initiating viral infection^29^. *TMPRSS2* expression facilitates SARS-CoV-2 S protein-mediated entry in human *ACE2*^+^ mature enterocytes in human small intestinal enteroids^30^. In the eye, high coexpression of *ACE2* and *TMPRSS2* has been observed in mouse cornea and human pterygium cell lines^31^, which may explain SARS-CoV-2 detection in the tears and conjunctival secretions of COVID-19 patients with conjunctivitis^32^. Thus, organ systems expressing host entry factors can explain extrapulmonary symptoms such as diarrhea and conjunctivitis.

Given the greater risk of mortality in COVID-19 patients with cardiovascular disease, regulation of *ACE2* in the cardiovascular system is a mechanism that may be involved. For instance, upregulated *ACE2* expression has been observed in the myocardium of COVID-19 patients with heart failure^33^, and single-nucleus RNA-seq (snRNA-seq) analysis has revealed higher *ACE2* expression in myocytes from patients with cardiomyopathy than in healthy controls. Under cardiac stress conditions, *ACE2* is repressed while *ACE* is induced, with consequent increased angiotensin II-mediated cardiac hypertrophy and fibrosis^34^. This response to cardiac stress in endothelial cells is the result of forkhead box M1 (FOXM1) promoter binding. In addition, regulation of *ACE2* in the cardiovascular system appears to be cell-specific, as *ACE2* was found to be expressed at low levels in cardiac fibroblasts but highly expressed in myocytes, macrophages and endothelial, and smooth muscle cells^35^.

Postmortem studies have provided direct evidence of viral RNA in the lungs and pharynx and, to a lesser extent, in the renal tubules, liver, heart, brain, and blood^36^. Similarly, endothelial dysfunction in postmortem lung and kidney tissue is associated with viral inclusion bodies, suggesting direct viral infection^37^. Hence, differential tissue expression of ACE2 and TMPRSS2 might link viral injury to organ systems and may explain the observed correlation between high respiratory and plasma viral loads and severe COVID-19 and mortality^38,39^. Other studies, however, have reported no association between viral load and disease severity^40^. This is supported by a systematic meta-analysis of viral load dynamics in COVID-19 whereby no viable virus was detected beyond day nine of the disease, despite prolonged SARS-CoV-2 RNA shedding in respiratory and stool samples^10^. Furthermore, a postmortem study demonstrated disconnect between SARS-CoV-2 detection and organ inflammation and dysfunction^41^. Together, these results suggest that disease progression and mortality may not be directly virus dependent but rather driven by aberrant host immune responses.

## Host factor expression associated with COVID-19 risk factors

Male sex has been independently associated with severe COVID-19 disease at presentation, the need for intensive care unit admission, and higher case fatality rates^42^. Differential host factor expression may explain the sex difference in COVID-19 (Fig. 1a, b), though the evidence is conflicting. A recent meta-analysis of scRNA-seq studies showed increased overall *ACE2* and *TMPRSS2* lung expression in males^43^; in contrast, others have found no significant sex difference with regard to *ACE2* expression^44,45^. Given the relatively low expression of *ACE2* and predominant AT2 cell expression, data for whole tissues compared to single-cell studies may also mask subtle differences. It is also noteworthy that while *ACE2* is located on the X chromosome, analysis of GTex data suggests that *ACE2* escapes X chromosome inactivation, resulting in sex-biased gene expression^46^. Sex differences in *ACE2* expression may also be due to regulation by hormone-dependent TFs. In female mice, *ACE2* basal activity in the kidney was repressed by estrogen, suggesting estrogen receptor (ER) response elements at the *ACE2* locus^47^. In the elderly human male atrial myocardium, ex vivo administration of estrogen induced *ACE2* mRNA through an ERα-dependent mechanism^48^. Although these findings support *ACE2* control via an ER response element, differences in the response might vary by tissue type, sex, age, and pathological state.

**Fig. 2:**
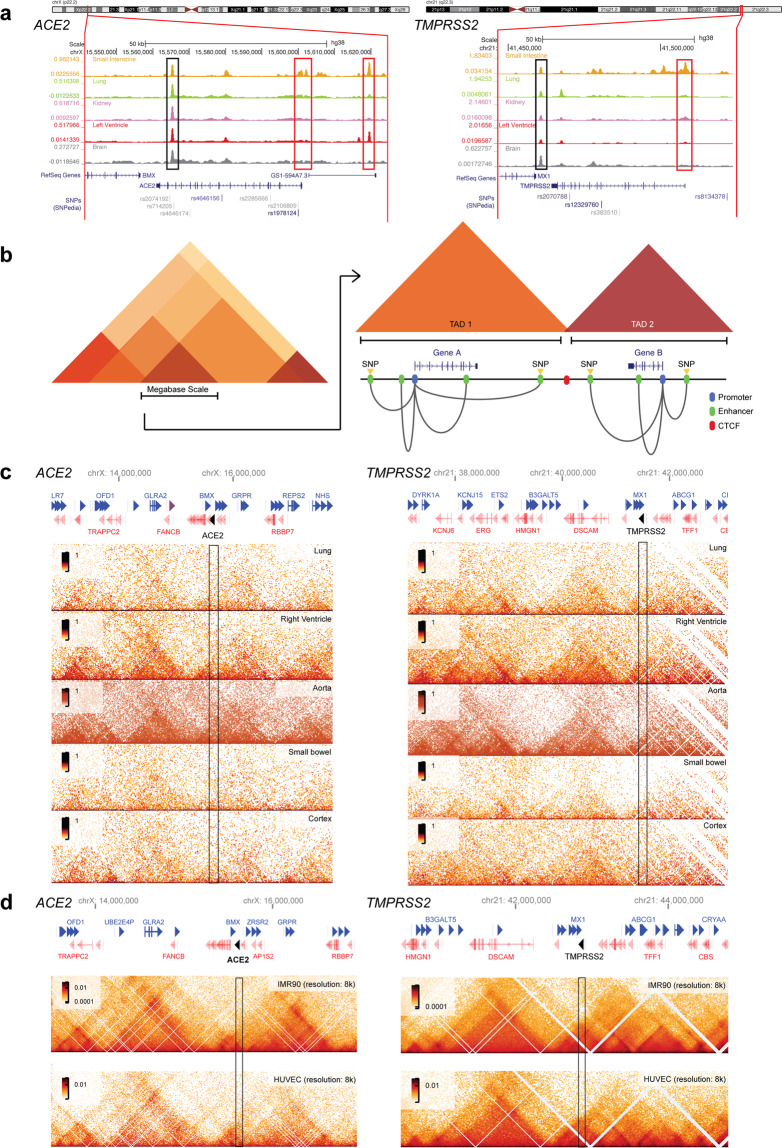
*ACE2* and *TMPRSS2* regulatory elements and tissue-invariant topologically-associated domains. **a** Human tissue DNase-seq signals for *ACE2* [left] and *TMPRSS2* [right] loci. Tissue-specific regulatory elements (red bar) and shared regulatory elements (black bar) are highlighted. Datasets were obtained from the Encyclopedia of DNA Elements (ENCODE) 2018 data release for Hg38^49^ and visualized via the UCSC genome browser. Normalized signal replicate pools are depicted for each tissue type. ENCODE experimental designations are ENCSR747YZZ (small intestine), ENCSR619JTC (lung), ENCSR955NXV (kidney), ENCSR871OSL (left ventricle) and ENCSR341MVE (brain). **b** Schematic representation of topologically-associated domains (TADs) showing boundaries for regulatory elements that control expression of the genes within a TAD. The formation of a TAD is dependent on insulator elements bound by CCCTC-binding factor (CTCF). Enhancer association with promoters via long-range interactions may act to regulate expression. Some enhancers harbor SNPs that might alter the expression pattern of genes within a TAD. **c** Hi-C data for human tissues depict chromosome conformation into TADs around *ACE2* (left) and *TMPRSS2* (right). The locations of the respective genes are indicated in black. Lung, right ventricle, small bowel, and cortex datasets were obtained from Schmitt et al.^94^. Aorta data were obtained from Leung et al.^108^. Hi-C datasets were visualized in HiGlass^109^ at 20-kb resolution. Datasets are aligned to human genome build Hg38. **d** Hi-C data for human lung fibroblast (IMR-90) and human endothelial (HUVEC) cell lines show higher-order chromosome organization as TADs. The genome locations of *ACE2* (left) and *TMPRSS2* (right) are indicated in black. Datasets aligned to human genome build Hg18 were obtained from Rao et al.^95^ and visualized in HiGlass^109^ at 8-kb resolution.

DNase-seq has revealed differential shared and tissue-specific regulatory element activation for the *ACE2* and *TMPRSS2* loci^49^ (Fig. 2a), likely regulated by cell-specific TF binding at regulatory elements^50^. ENCODE datasets show binding of the insulator CCCTC-binding factor (CTCF) at shared sites for *ACE2* and *TMPRSS2*, whereas activator protein-1 (AP-1) subunits bind to the tissue-specific 5′ upstream element of *ACE2*, suggesting that variable host factor gene expression may be explained by differential engagement of regulatory elements and selective TF binding.

Regulation of *TMPRSS2* has primarily been explored in prostate cancer. In this context, *TMPRSS2* is induced by androgens through a distal androgen receptor (AR) binding enhancer^51^, resulting in AR-induced expression of a *TMPRSS2-ERG* (ETS-related gene) fusion gene conferring prostate cancer pathogenicity^52^. Mapping of an AR response element to the induction of *TMPRSS2* expression has led to the hypothesis that androgenic phenotypes are risk factors for COVID-19^53^. Epidemiological studies have also suggested androgenic alopecia as a separate risk profile for COVID-19^54^. However, AR blockade in mice did not alter lung *TMPRSS2* expression^55^, suggesting that lung *TMPRSS2* may not be androgen regulated, possibly through tissue-specific enhancer inaccessibility. As *TMPRSS2* promoters in mammary epithelial cells have been found to be responsive to estrogens^56^, the role of *TMPRSS2* in conferring risk in men through sex-specific expression in relevant tissues remains unclear.

Moreover, sex-biased behavior may contribute to male predominance of COVID-19. Higher rates of smoking among men have been hypothesized to contribute to male predisposition towards the disease^57^, and active smoking is implicated as a negative prognostic indicator for COVID-19^24^. This would be consistent with increased *ACE2* in the bronchial epithelium^58^ and small airway epithelium of chronic smokers^45,59^. scRNA-seq data showed dose-dependent upregulation of *ACE2* by cigarette smoke in rodent and human lungs, with chronic smoke exposure triggering an expansion of *ACE2*^+^ mucus-secreting goblet cells^60^.

Age-related dynamics in host factor expression may similarly explain the greater severity and mortality that occurs among older patents^9,61^. Indeed, older age is associated with higher viral load in oropharyngeal salivary and respiratory samples^62^. Lung *ACE2* expression has been shown to increase ~1.2-fold with every 10-year increase in age^44^. scRNA-seq of developing mouse lungs and temporally resolved RNA-in situ hybridization (ISH) analysis also found an age-related increase in *TMPRSS2* expression^63^. Single-nucleus ATAC-seq (snATAC-seq) in human AT2 cells demonstrated age-associated changes in accessible chromatin around *TMPRSS2* at sites harboring sequence motifs for multiple TFs, including forkhead box A (FOXA), C/EBPα, the proinflammatory factors STAT, IFN-regulatory factor (IRF), and AP-1, suggesting age-dependent inflammatory regulation of *TMPRSS2*^64^. High ACE2 and TMPRSS2 levels in the elderly may therefore be associated with increased viral infection, although altered immune response and physiology may also explain divergence in severity with age extremes.

Although lower nasal epithelial *ACE2* expression reported in children^65^ has been suggested to explain the milder disease observed in this population^66^, others have found that pulmonary ACE2 is not reduced in children^13^. Furthermore, scRNA-seq and immunohistochemistry of human respiratory tract tissue indicated no significant difference in ACE2 localization in those with independent risk factors for severe COVID-19, such as male sex, asthma, cardiovascular disease, COPD, diabetes, and smoking, compared to age-matched healthy controls^1,2,13^. This contradictory evidence suggests a complex relationship between pulmonary ACE2 expression and COVID-19 as both a viral receptor and protective factor, implicating other host factors in regulating disease severity and susceptibility.

## Host genetic variants and the spectrum of COVID-19

Host genetic factors have been implicated in influenza and zoonotic diseases such as SARS and MERS^67,68^. Genetic variants may alter disease through viral infection, replication or immune function by altering host gene expression or protein function, leading to heterogeneous severity.

### Genetic variants of ACE2 and TMPRSS2

**Fig. 3:**
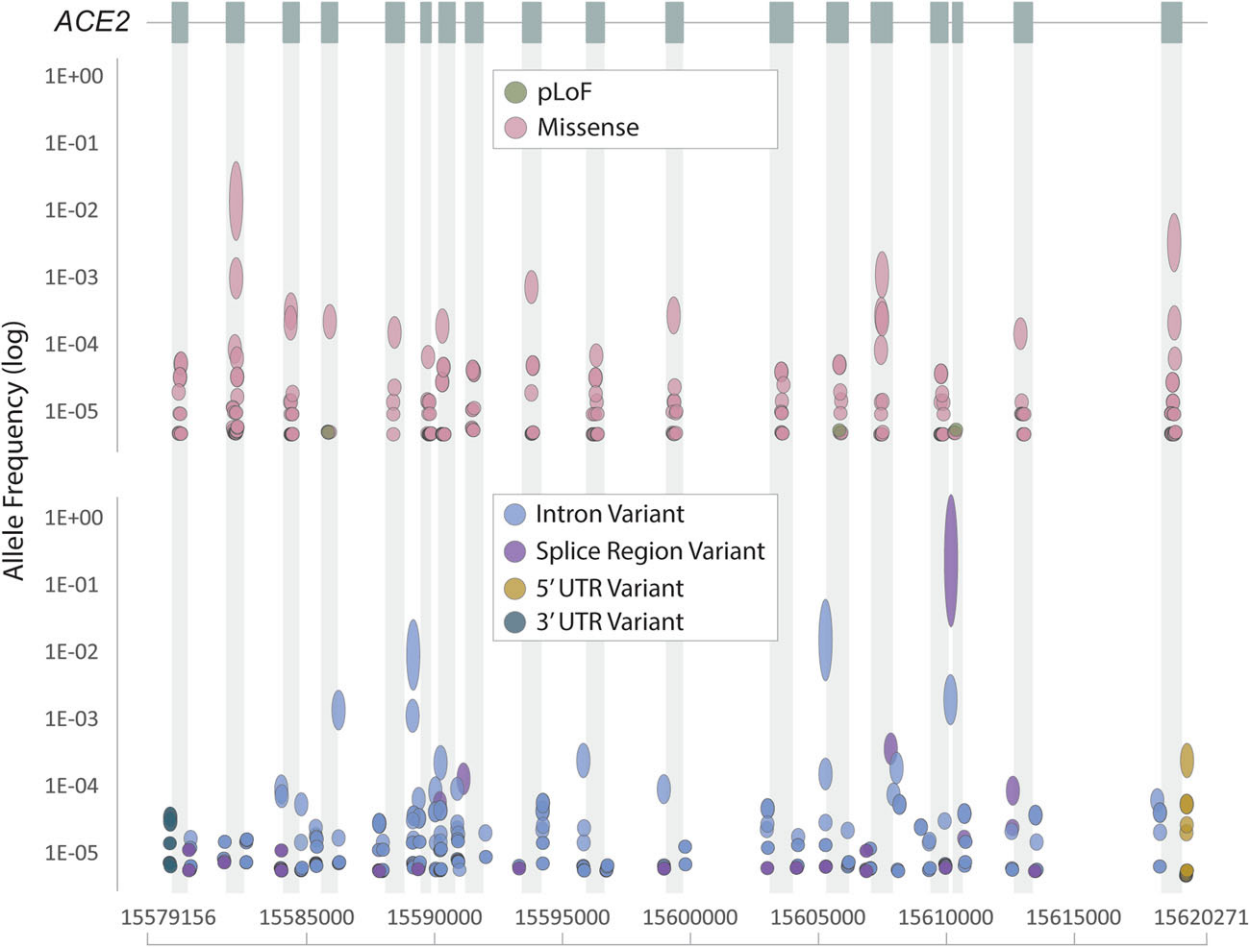
Single-nucleotide polymorphism (SNP) allelic frequencies of angiotensin-converting enzyme 2 (ACE2). *ACE2* (chromosome X: 15579156–15620271) exon coding transcripts are represented by boxes and corresponding shaded areas. Predicted loss-of-function (pLOF) and missense variants (top panel) and intron, splice region, 5’ untranslated region (UTR), and 3’UTR variants (bottom panel) are shown. The size of the dot reflects the minor allele count (range: 1-45,478), with minor allele frequency represented on the *y*-axis. Data were obtained from gnomAD v2.1.1^69^.

Human variants of *ACE2* could alter the protein interface with the SARS-CoV-2 S protein. Moreover, as *ACE2* is located on the X chromosome, hemizygosity for harmful variants might contribute to poor outcomes in men with COVID-19. Genomic variant analysis from Genome Aggregation Database (gnomAD)^69^ reveals a very low frequency of non-synonymous *ACE2* variants, including those predicted to be loss-of-function (Fig. 3). Furthermore, no correlation between rare deleterious *ACE2* exonic and splice junction variants with COVID-19 was observed in an Italian population^70^.

Given the role of ACE2 in cardiovascular disease and lung disease, including ARDS, *ACE2* polymorphisms are of interest beyond COVID-19. For example, a common exonic SNP in *ACE2*, rs2285666 (Fig. 2a), has been implicated in the risk of cardiovascular disease and type 2 diabetes mellitus, with the rare A allele found at a frequency of 0.2% in European populations and 0.55% in East Asians^70^. Two *ACE2* intronic SNPs, rs2074192 and rs2106809, associated with left ventricular hypertension are expression quantitative trait loci (eQTLs) for *ACE2* and may therefore link *ACE2* cardiac expression with high penetrance of alleles, increasing *ACE2* expression in East Asians^61^. *ACE2* variants could therefore be linked to risk factors for COVID-19, but further clarification within comorbid subgroups is required.

For *TMPRSS2*, the frequency of rare deleterious and common exonic variants speculated to alter protein levels or activity varies between populations. For common variants, two haplotypes have been identified. One is linked to an eQTL harboring rs8134378 and mapping to an AR regulatory element 15 kb upstream of the *TMPRSS2* CpG island^70^. The second includes the intronic SNP rs2070788 (Fig. 2a), which has been found in small-scale genome-wide association studies (GWASs) to be linked to an increased risk of influenza^71^. Less is known about *TMPRSS2* variants, warranting further investigation. However, in the first two published GWASs for severe COVID-19, there was no significant association with *ACE2* or *TMPRSS2* variants, suggesting that variants of these factors do not contribute to COVID-19 severity^3,72^.

### Genetic variants in intracellular RNA sensing and type I interferon signaling

Based upon the gene mutations implicated in life-threatening influenza pneumonia, rare but predicted deleterious mutations have been examined in patients with severe COVID-19^72^. Mutations in the genes encoding double-stranded RNA sensor TLR3 and in downstream components of type I IFN cell-intrinsic immunity have been detected, including UNC93B1, which forms a complex with and is involved in intracellular trafficking of TLR3^73^ and other downstream components, such as TRIF and TBK1, a key kinase linking pattern recognition receptor activation and activation of ISGs. Mutations in the type I IFN amplification pathway were also found, including in the genes coding for IRF7 and the cell surface receptors IFNAR1 and IFNAR2. In addition, common variants at *IFNAR2* were found in a GWAS for severe COVID-19^3^. Although no rare variants in the JAK/STAT pathway downstream of IFNAR1/2 have been reported in severe COVID-19, variants at *TYK2* on chromosome 19 were reported in the GWAS of a UK intensive care cohort of COVID-19 patients with critical respiratory failure^3^. Of note, patients with complete TYK2 deficiency are susceptible to bacterial and viral infection^74^. The contribution of genetic variants to reduced IFN signaling in severe COVID-19 is further supported by observations of impaired IFN responsiveness in neutrophils from patients with severe COVID-19^75^. Nonetheless, the WHO Solidarity Trial has shown that treatment of COVID-19 patients with IFN-β1a did not decrease mortality or the need for mechanical ventilation^76^.

Some critical COVID-19 patients with mutations in IFN pathways exhibit reduced serum IFN-α^72^, though other studies have reported higher IFN-γ in helper T cells of patients who died from COVID-19^77^. IFN-γ is linked to IL-6 signaling through the IL-6 receptor (IL6R)^78^, suggesting interplay between IFN and interleukin signaling in acute inflammation. Genetic variants of IL6R are associated with a lower risk of hospitalization following SARS-CoV-2 infection^79^, yet these variants were not significant in the COVID-19 GWASs above. Nevertheless, modulation of the IL6R axis using inhibitors, such as tocilizumab and sarilumab, was shown in the REMAP-CAP and EMPACTA trials to reduce the need for mechanical ventilation and reduce mortality in patients with severe COVID-19^80,81^.

**Fig. 4:**
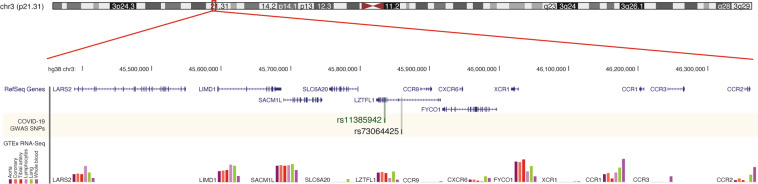
The 3p21.31 locus harbors significant single-nucleotide variants from the COVID-19 GWAS. The COVID-19 GWAS identified SNPs mapping to the 3p21.31 locus. The lead SNP from each study (rs11385942 from Ellinghaus et al.^82^; rs73064425 from Pairo-Castineira et al.^3^) mapped to *LZTFL1*. Images were generated from the UCSC genome browser (hg38, chr3:45,388,000–46,361,000), showing RefSeq genes and Genotype-Tissue Expression (GTEx) RNA-seq data for selected tissues^107^.

In the first published GWAS for severe COVID-19, a significant cross-replicating locus at 3p21.31 was found in Italian and Spanish populations^82^, spanning six genes: *LC6A20*, *LZTFL1*, *CCR9*, *FYCO1*, *CXCR6*, and *XCR1* (Fig. 4). The insertion–deletion GA risk variant of rs11385942 in an intron of *LZTFL1* is associated with reduced *CXCR6* but increased *SLCA20* expression. Interestingly, the risk allele was detected frequently in patients who required mechanical ventilation, and furthermore, the few individuals homozygous for the risk allele were younger, suggesting an association with COVID-19 severity. The frequency of risk allele heterozygosity varies between populations, occurring in less than 0.5% in China and 37.8% in Bangladesh^82^. Moreover, a GWAS of the UK intensive care cohort of COVID-19 patients with critical respiratory failure^3^ replicated the significance of the chromosome 3 locus.

The genes at the 3p21.31 locus have plausible roles in COVID-19 and lung injury. *SLC6A20* encodes a sodium-dependent amino transporter that has been shown to interact with ACE2 in a Xenopus oocyte expression system^83^. CXCR6 is a marker of lung-resident memory CD8^+^ T cells, producing cytokines in response to stimuli^84^^.^ Its ligand, CXCL16, is elevated in the serum of patients with acute lung injury, and both ligand *CXCL16* and *CXCR6* receptor genes are upregulated in models of lung injury^9^. CXCL16-CXCR6 acts to disrupt epithelial integrity through downregulation of E-cadherin in lung injury^9^, which might represent the fibrosis observed in ARDS. CCR9, as part of a CCL25-CCR9 chemokine axis, has been shown to mediate gut-specific recruitment of lung IFN-γ-producing CCR9^+^CD4^+^ T-cells in influenza, contributing to the gut injury observed in viral infection^64^. *FYCO1*, encoding an autophagy adaptor protein, was found to be upregulated in intensive care patients with sepsis^85^.

The second locus uncovered by Ellinghaus et al. maps to the *ABO* blood group gene, whereby group A confers a higher risk of severe COVID-19 and group O demonstrates a protective effect^82^. The plausibility of an association is supported by an epidemiological study in a Chinese population, showing increased COVID-19 risk with blood group A and reduced risk with blood group O^86^. However, the association of ABO blood groups with COVID-19 severity was not replicated by the GenOMICC study^3^. Additional loci associated with COVID-associated ICU admission include the *OAS* gene cluster on chromosome 12 and *DPP9* on chromosome 19^3^. *OAS* genes are activated by IFN and are involved in double-stranded RNA degradation pathways in the early innate antiviral response^87,88^. *DPP9* encodes a serine protease implicated in lung fibrosis^89^. These pathways may therefore represent therapeutic targets to mitigate progression to critical respiratory failure in COVID-19.

### Mechanisms of genetic variants driving increased disease risk

Most GWAS disease-risk loci are in noncoding regions of the genome^90^. The cataloging of human regulatory elements on a genome-scale across multiple cell and tissue types has uncovered a correlation between accessible chromatin, DNA-binding factors and SNPs^91^. These large-scale studies implicate altered TF binding as a core feature of noncoding genetic variants. For example, in lymphoblastoid cell lines, interindividual variation in NF-kB binding has been associated with genetic variants and alterations in gene expression^92^. Genetic variation could, therefore, alter responses to stimuli, such as infection, between individuals and thus influence severity.

To infer phenotypic consequences of genetic variants, functional assignment of genetic variants to gene regulation is required but remains challenging. Chromatin structure can be used to aid functional characterization of variants. Chromatin higher-order structures have been found to form topologically-associated domains (TADs), regions of chromatin that show high interaction frequencies separated by boundary regions^93^ (Fig. 2b). At the megabase scale, TADs are generally stable between cell types and tissues^94,95^. TAD boundaries are marked by binding sites for CTCF, which impedes cohesin-driven loop extrusion, the process responsible for TAD formation. For the most part, enhancers are generally found in the same TAD as the gene(s) they regulate^96^. Therefore, incorporating information on chromatin structure, including smaller scale accessibility information with larger scale TADs, may aid in the discovery of associations between noncoding variants and genes to guide fine-mapping experiments^97^ and has allowed functional refinement of SNPs and eQTLs^98^.

*ACE2* and *TMPRSS2* are located within tissue-conserved TADs (Fig. 2c, d). However, eQTL analysis of SNPs at the *ACE2* locus did not reveal an association with gene expression in lung tissue^99^. Within the *ACE2* TAD (hg38 chrX:15,300,000–15,600,000), long-distance interactions were observed between multiple SNPs and other genes associated with eQTLs in the lung, such as *PIR*, a modulator of NF-kB signaling, and *CA5B*, which is involved in acid-base metabolism^99^.

Incorporating open chromatin might also contribute to the identification of functional genetic variants. By applying snATAC-seq to healthy human lungs, age-dependent accessible regions in *TMPRSS2*-expressing AT2 cells were found to be enriched for genes associated with inflammatory signals^100^. In AT2 cells, age-dependent accessible chromatin near *TMPRSS2* correlated with sequence variants, including common variants with a minor allele frequency >1%^100^. These variants are predicted to disrupt sequence motifs of DNA-binding factors and thus alter expression and are associated with GWAS phenotypes for emphysema, asthma, and bacterial pneumonia^100^.

## Conclusion

The sequalae following SARS-CoV-2 infection are highly heterogeneous, from asymptomatic to multiorgan failure, and host factors are likely variables involved in COVID-19 severity^101^. Risk factors for COVID-19 contribute to modified disease risk through changes in viral–host interactions, most likely through host factor expression. ACE2 and TMPRSS2 have been identified as SARS-CoV-2 tropic factors. However, ACE2 was found to be protective against pulmonary injury through maintenance of vascular competence in mouse models of acute lung injury^102^. The balance between viral exploitation of host factors and the role of that factor in COVID-19 pathophysiology is not clear. Furthermore, *ACE2* and *TMPRSS2* genetic variants are not convincingly associated with COVID-19 severity^3,82^. Other important host factors involved in viral–host interactions, viral protein processing, and immune function are more likely to contribute to severity. The therapeutic effect of glucocorticoids, such as dexamethasone^103^ or hydrocortisone^104^, in decreasing COVID-19 mortality or the need for organ support strongly suggests an inflammatory process in severe disease. IFN pathways are also likely to play key roles in COVID-19, and their impairment might explain progression to critical illness. While the WHO Solidarity trial does not support the use of IFN as a treatment modality^76^, questions on the timing and regime remain while highlighting a need for alternate targets. Exploring the expression variation and regulatory control of other host factors, such as the 3p21.31 locus identified from COVID-19 GWASs, will aid in our understanding of the disease spectrum and uncover therapeutic targets^3,82^. It will also be important to integrate comorbidities with the genetic variants implicated in COVID-19 to understand the contributions of risk factors to COVID-19 severity.

The identification of functional regulatory elements in health and pathological states is a crucial pursuit, with many approaches being developed following the mapping of noncoding genomic regions. We have highlighted a number of methods that have been employed to identify regulatory elements, but their use in conglomerate is likely required, taking a *multiomics* approach to identify functional consequences. This is particularly important for understanding dynamic changes in regulatory control, for instance, during inflammation. The development of single-cell methodologies, as an example, has also revolutionised our discovery and understanding of cell types and heterogeneous RNA expression in complex tissues. Nevertheless, functional studies of complex tissues have been largely limited to model organisms, with questionable translational potential. The development of human organoid systems may allow for a more relevant understanding of the molecular processes that drive variation in disease presentation and progression. Combining these approaches with functional assays for regulatory control of gene expression by using, for example, high-throughput CRISPR-based perturbation methods^105^ or a high-throughput self-expressing reporter system to identify functional enhancer elements in cell-specific control^106^, would allow dissection of the roles of regulatory elements, genetic variants or biological pathways in disease processes such as viral infection. Such approaches will allow the identification of variables to allow risk stratification for public health initiatives, resource allocation for at-risk groups and the development of novel therapeutics.
